# Supplementary material for: Who Seeks Help from Live Chat Services? Demographic, Psychosocial, and Service Use Profiles of Young People Using a National Live Chat Mental Health Service
Source: Glob Ment Health (Camb). 2026 Jan 22;13:e32. doi: 10.1017/gmh.2025.10120 (PMC12951336; doi:10.1017/gmh.2025.10120)
Supplement: Tibbs and Fitzgerald supplementary material [file S2054425125101209sup001.docx]

**Supplementary Materials**

Table 1
*Clinical Cut-offs for YP-CORE*

| Clinical Score | Gender | Age |
| --- | --- | --- |
| 10.3 | Male | 11-13 |
| 14.1 | Male | 14-16 |
| 14.4 | Female | 11-13 |
| 15.9 | Female | 14-16 |

*Note.* The YP-CORE is completed by 12–16-year-olds and the CORE-10 by 17–25 -year-olds. A cut-off to denote the clinical range of distress has not yet been determined for gender-diverse populations.

Table 2
*Definitions of key Live Chat engagement metrics.*

| **Variable** | **Definition** |
| --- | --- |
| Chat duration | The time elapsed between the first and last message is sent. |
| Wait time | Time (in minutes) elapsed between entering the queue and accessing the chat interface. |
| Dropout/unsuccessful chat | Chat sessions where the service user exited the wait queue before entering the chat interface or chat sessions where the service user drops off chat before sending at least one chat message. |
| Service user response latency | Time (in minutes) taken to respond to previous clinician chat message. |
| Clinician response latency | Time (in minutes) taken to respond to previous service user chat message. |
| Chat duration | Time (in minutes) elapsed between the first and last chat message. |
| Service satisfaction | Measured by an adapted version of the eheadspace satisfaction questionnaire (Rickwood et al., 2019). |

Table 3
*Example of inductive coding approach*

| **Reason for Attendance** | **Meaning Unit** | **Code** | **Category** | **Thematic Area** |
| --- | --- | --- | --- | --- |
| “don't know how to feel argued with a boy no appetite serious overthinking with everything” | Argued with boy | Issues in romantic relationships - conflict | Relationship issues | Presenting Mental Health Difficulties |
|  | No appetite | Eating/appetite issues | Somatic issues |  |
|  | Serious overthinking with everything | Overthinking | Maladaptive or negative thought patterns |  |

Table 4
*Youth Reported Presenting Mental Health Difficulties.*

| **Presenting Mental Health Difficulty** | **N(%)** |
| --- | --- |
| **Anxiety** | **365 (22.4)** |
| Generalised/unspecified | 246 (67.4) |
| Context-specific | 46 (12.6) |
| Panic/panic attacks | 39 (10.7) |
| Social | 24 (6.6) |
| Health | 7 (1.9) |
| Fear/phobia | 3 (0.8) |
| **Low Mood** | **278 (17.1)** |
| Feeling sad/down/low | 157 (56.5) |
| Feeling depressed | 67 (24.1) |
| Lack of energy/motivation | 39 (14.0) |
| Hopelessness | 12 (4.3) |
| Isolating self | 3 (1.1) |
| **Relationship Issues** | **189 (11.6)** |
| Relationships with family | 72 (38.1) |
| Romantic relationships | 55 (29.1) |
| Friendships | 40 (21.2) |
| Generalised/unspecified | 16 (8.5) |
| Bullying | 6 (3.2) |
| **General Psychological Distress** | **154 (9.4)** |
| Generalised/unspecified | 66 (42.8) |
| Feeling overwhelmed | 45 (29.2) |
| Feeling lost/confused | 20 (13.0) |
| Detachment from reality/feeling numb | 12 (7.8) |
| Problems focusing | 7 (4.6) |
| Feeling guilty | 4 (2.6) |
| **Stress** | **89 (5.5)** |
| Context-specific | 46 (51.7) |
| Generalised/unspecified | 43 (48.3) |
| **Loneliness or Lack of Social Support** | **84 (5.2)** |
| **Negative self-perception** | **73 (4.5)** |
| Self-Esteem | 50 (68.5) |
| Body Image | 23 (31.5) |
| **Risk** | **70 (4.3)** |
| Self-Harm | 38 (54.3) |
| Suicidal Ideation | 26 (37.1) |
| Risk to Others | 4 (5.7) |
| Suicide Attempt | 2 (2.9) |
| **Maladaptive or negative thought patterns** | **60 (3.7)** |
| Overthinking/rumination | 27 (45) |
| Negative thoughts | 14 (23.3) |
| Intrusive thoughts | 14 (23.3) |
| Paranoia | 5 (8.3) |
| **Eating Concerns/Issues** | **38 (2.3)** |
| **Emotion Regulation** | **36 (2.2)** |
| Anger | 19 (52.8) |
| Mood fluctuations/controlling emotions | 17 (47.2) |
| **Problems in school/college** | **33 (2.0)** |
| **Identity issues** | **32 (1.9)** |
| Gender Identity | 18 (56.2) |
| Sexual Identity | 10 (31.3) |
| Other | 4 (12.5) |
| **Somatic issues** | **30 (1.8)** |
| Sleep changes | 21 (70) |
| Appetite changes | 7 (23.3) |
| Physical pain | 2 (6.7) |
| **Concerns about the pandemic** | **26 (1.6)** |
| Psychological | 23 (88.5) |
| Loneliness/Isolation | 3 (11.5) |
| **Experiences of Trauma or exposure to traumatic events** | **19 (1.2)** |
| General/Unspecified | 11 (57.9) |
| Sexual Abuse/Assault | 5 (26.3) |
| Physical Abuse | 3 (15.8) |
| **Bereavement and Loss** | **15 (0.9)** |
| **Disorder specific issues** | **11 (0.7)** |
| OCD | 4 (36.4) |
| BPD | 3 (27.3) |
| ADHD | 2 (18.2) |
| Autism | 1 (9.1) |
| Bipolar | 1 (9.1) |
| **Substance use/addiction** | **10 (0.6)** |
| **Financial, work, and housing-related concerns** | **9 (0.5)** |
| **Physical Health** | **6 (0.3)** |
